# Painting a new picture of personalised medicine for diabetes

**DOI:** 10.1007/s00125-017-4210-x

**Published:** 2017-02-07

**Authors:** Mark I. McCarthy

**Affiliations:** 1 Oxford Centre for Diabetes, Endocrinology and Metabolism, University of Oxford, Churchill Hospital, Old Road, Headington, Oxford, OX3 7LJ UK; 2 Wellcome Trust Centre for Human Genetics, University of Oxford, Oxford, UK; 3 Oxford NIHR Biomedical Research Centre, Oxford University Hospitals Trust, Oxford, UK

**Keywords:** Biomarkers, Complex disease, Diabetes, Environment, Genetics, Pathogenesis, Review, Risk, Taxonomy

## Abstract

The current focus on delivery of personalised (or precision) medicine reflects the expectation that developments in genomics, imaging and other domains will extend our diagnostic and prognostic capabilities, and enable more effective targeting of current and future preventative and therapeutic options. The clinical benefits of this approach are already being realised in rare diseases and cancer but the impact on management of complex diseases, such as type 2 diabetes, remains limited. This may reflect reliance on inappropriate models of disease architecture, based around rare, high-impact genetic and environmental exposures that are poorly suited to our emerging understanding of type 2 diabetes. This review proposes an alternative ‘palette’ model, centred on a molecular taxonomy that focuses on positioning an individual with respect to the major pathophysiological processes that contribute to diabetes risk and progression. This model anticipates that many individuals with diabetes will have multiple parallel defects that affect several of these processes. One corollary of this model is that research efforts should, at least initially, be targeted towards identifying and characterising individuals whose adverse metabolic trajectory is dominated by perturbation in a restricted set of processes.

**Electronic supplementary material:**

The online version of this article (doi:10.1007/s00125-017-4210-x) contains a slideset of the figures for download, which is available to authorised users.

## Personalised medicine for the characterisation of disease

The science of medicine is focused around notions of categorisation. Most obviously, we categorise the ways in which people become unhealthy through the application of diagnostic labels [1]. In doing so, we hope to co-locate the diseased individual with others who share similar pathology so that we can gather, through that similarity, clues to their prognosis and the ways in which we might most efficiently intervene to minimise further harm. These goals are not new; most readers of this journal will be familiar with the claims that, 1500 years ago, Indian physicians had figured out the clinical distinction between what we now term type 1 and type 2 diabetes [2].

Such concepts have been given a new airing in recent years, with growing interest and investment in delivering what has been termed personalised or, more or less equivalently, precision medicine [3]. The rationale is that current ways of categorising disease are outmoded and, for many diseases, have failed to achieve their essential objectives. Individuals who share the same diagnostic labels may have very heterogeneous presentations and clinical courses, and respond very differently to the suite of available lifestyle or therapeutic interventions. The expectation driving personalised medicine is that a swath of transformative technologies that allow a far more detailed dissection of in vivo pathology than hitherto possible (including, but not limited to, DNA sequencing, functional imaging and a range of ‘omic’ measurements) will provide the basis for ever more exquisite refinement of disease subtype, and support effective optimisation of disease management to match individual pathology.

## Promises and challenges in personalised medicine

There is increasing evidence to support this approach [4]. In rare (monogenic) diseases, DNA sequencing has characterised many causal genes, defined novel syndromes, highlighted new treatments and curtailed the traumatic diagnostic odyssey of many families. In cancer, tumour sequencing represents an increasingly routine component of care and there have been notable successes (sadly, often only short-lived) in using this information to select bespoke medications. Elsewhere, testing of genetic variants that increase the risk of severe adverse drug events (e.g. Stevens–Johnson syndrome) offers a path to safer therapeutic choices.

We are clearly at the start of something that will transform many aspects of medicine. At the same time, for most diabetologists, the promise that genetics, genomics and imaging will usher in a step change in our ability to deliver personalised medicine may seem distant and irrelevant. Indeed, the translational impact of these technologies on many of the complex, multifactorial conditions that, like type 2 diabetes, afflict individuals in later life remains minimal.

It is worth starting to understand why this might be by noting what it is that distinguishes rare disease, pharmacogenetics and cancer (the initial foci of personalised medicine success) from a disease like type 2 diabetes. In the case of rare diseases and pharmacogenetics, the causal event (a mutation or a drug) is uncommon, precise and has high phenotypic impact, typically resulting in an extreme phenotype that is qualitatively distinct and easy to recognise. Successful examples of personalised medicine in cancer often rest on the direct detection of penetrant somatic mutations in the diseased tissue. In contrast, type 2 diabetes features multiple, common, low-impact risk variants, pervasive environmental exposures and a phenotype that very clearly lies on a quantitative spectrum of metabolic disturbance. Furthermore, diseased tissue is rarely accessible [5, 6].

## The prevailing diagnostic model for diabetes

How then should we envisage ‘personalisation’ of medicine for a complex, multifactorial disease like diabetes?

In this context, it is commonplace to hear comments such as ‘type 2 diabetes is highly heterogeneous’ or ‘type 2 diabetes is really a mixture of diseases’. The implication is that, at some future date, the disease that we currently describe as ‘type 2 diabetes’ will have been fragmented into a series of distinct diagnostic categories (we could call them type 2A, type 2B…type 2Z). This would be analogous to the subtypes of anaemia (Fe-deficient, vitamin B_12_-deficient, thalassaemia etc.), which reflect distinct pathological processes.

It also evokes the molecular classification of monogenic diabetes. Where two decades ago we had categories defined on clinical grounds (such as MODY, and permanent and transient forms of neonatal diabetes mellitus), we now have a molecular taxonomy of distinct diseases, many of which are associated with precise prognostic and therapeutic consequences [7].

For the purposes of easy distinction from the alternative that I propose later, let us call this the ‘pigeonhole’ model, based on the notion that it will allow the clinician to locate each diabetic patient to their appropriate diagnostic cubby-hole: for example, ‘You have type 2F diabetes; your diabetes is caused by a defect in *x* and the correct treatment is *y*’ (see Table 1).

**Table 1:** Distinctive features of the ‘pigeonhole’ and ‘palette’ models

Features	Pigeonhole model	Palette model
Description	Discrete diagnostic categories based on the individual clinical picture	Blended phenotypic characterisation based on the contribution of component pathways to individual pathology
Architecture	Most consistent with rare, high-impact genetic and environmental exposures	More consistent with multiple, parallel and common genetic and environmental risk factors
Heterogeneity	Assumes relative homogeneity within diagnostic groups	Assumes heterogeneity arising from a multiplicity of contributing processes is the rule, not the exception
Temporal	Envisages fixed diagnostic labels	Consistent with evolution of the clinical picture over time
Primary/reactive	Assumes the phenotype is dominated by primary impacts	Recognises the impact of both primary and reactive processes
Diagnostic focus	Undue focus on assigning individuals who span diagnostic boundaries to the ‘correct’ diagnosis	Highlights focus on individuals (‘archetypes’) with less complex aetiologies

I have three principal objections to this perspective for a sustainable diagnostic taxonomy for diabetes. First, it is increasingly clear that individual predisposition to diabetes is influenced by multiple independent genetic and non-genetic factors. As we, and others, have shown, the bulk of the genetic predisposition for type 2 diabetes resides in a large number (several thousands) of mostly common genetic variants [5]. Each of us carries a ‘barcode’ of risk and protective alleles across these variants that is individually unique, but, at the same time, in no way distinctive. And whilst we have a poor understanding of the specific environmental exposures that have the greatest influence on diabetes risk, the global surge in diabetes prevalence indicates that these are also likely to be pervasive [6]. Each of us ages within a miasma of common, shared and overlapping genetic and environmental factors that tune our individual risk of type 2 diabetes in ways that are very different from the rare, high-impact alleles that determine monogenic phenotypes.

Second, the pigeonhole model of diabetes simply does not work. Whilst type 1 and type 2 diabetes appear to reflect broadly different processes, distinguished largely by the contribution of islet autoimmunity to their pathogenesis, even here the distinction is far from absolute. A non-trivial proportion of the genetic variants shown to influence type 1 diabetes also influence type 2 risk, some of them in a coordinated direction (the same allele increases risk for both types of diabetes), others in opposite directions (the allele that increases type 1 diabetes risk reduces type 2 risk) [8]. Further, because the predispositions to develop islet autoimmunity and type 2 diabetes are both common, many individuals are at increased risk of both of these conditions. The genetic profiles of such individuals reveal a mix of type 1 and type 2 diabetes risk alleles, and the diabetes that they develop overlays evidence of autoimmunity on top of a type 2 phenotype [9]. These individuals fit poorly into either parent category and have been granted their own diagnostic label (latent autoimmune diabetes of adulthood), albeit one with indistinct and poorly-defined boundaries [10].

To cite another example of diagnostic overlap, whilst there are clearly some families in which mutations of *HNF1A* (or other MODY genes) have a powerful impact on the presentation of diabetes (otherwise linkage analysis would never have succeeded in mapping the causal genes), the overall phenotypic impact of those variants is less extreme than has been generally assumed. Many of the alleles considered to be causal for MODY are also observed in individuals who, on clinical grounds, have what would otherwise be considered late-onset type 2 diabetes or, indeed, entirely normal glucose profiles into later life [11]. The boundaries between monogenic and common forms of diabetes are not as clear as we like to think [5, 12].

Third, whilst there is no doubt that the existing categories have clear value in picking out some of the key processes involved in diabetes pathogenesis and in defining appropriate therapy (no one argues with the use of replacement insulin in those whose beta cells have been destroyed), there comes a point at which the expectation that all patients can be assigned to one or other diagnostic category becomes self-defeating. A disproportionate amount of effort goes into dealing with the many individuals whose clinical and molecular picture resists such facile categorisation [13].

The problem lies in taking a model that works well for rare monogenic diseases (and, to some extent, for cancer), where the categories reflect distinct and discrete pathologies, and trying to apply it to common complex diseases that are, in terms of their diagnostic architecture, very different. Others have reached similar conclusions. For example, a decade ago, in this journal, E. Gale railed with customary philosophical panache against the reification of type 1 and type 2 diabetes as distinct diagnostic entities [14]. More recently, Schwartz and colleagues detailed their concerns regarding the ‘conflicting and confounding definitions’ of existing diabetes categories [13].

## An alternative diagnostic model for diabetes

**Fig. 1:**
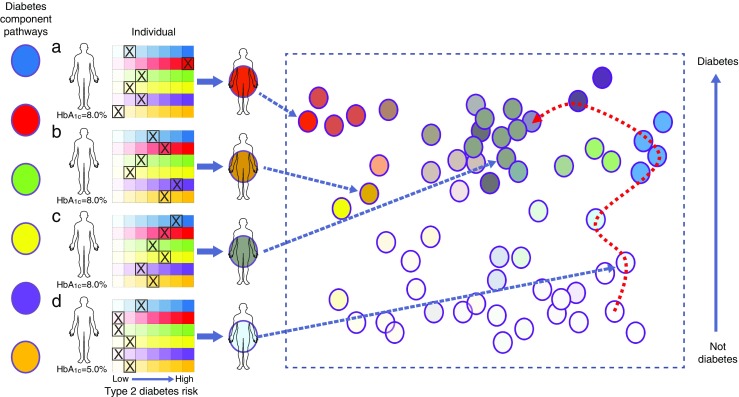
The ‘palette’ model of type 2 diabetes. The concepts are illustrated using a model of six diabetes component pathways (‘base colours’) and four individuals, three of whom have diabetes. The grids display the range of trait variation for each of these component pathways and the position of each individual on each of those spectra. The pathophysiology of individual ‘a’ is dominated by a single process (shown in red), that of individuals ‘b’ and ‘c’ reflects contributions from multiple processes (resulting in an aggregated brown or grey colour, respectively). Individual ‘d’ does not have diabetes but shows type 2 diabetes-associated risk in the blue process. To the right, data from a larger cohort have been used to position individuals in a multidimensional space (reduced to two dimensions here for illustrative purposes) with respect to the status of each of the component pathways (with hue denoting the mixture of type 2 diabetes-associated contributions and saturation broadly reflecting diabetic status). Some individuals, such as individual ‘a’, lie at the extremes and represent ‘archetypes’ for particular component pathways, whereas individuals ‘b’ and ‘c’ lie more centrally, reflecting a more complex pathophysiology. Individual ‘d’ is currently not diabetic but the red dotted line describes their past and future path through this multidimensional space, up to and beyond the point at which diabetes is diagnosed. To convert values for HbA_1c_ in % into mmol/mol, subtract 2.15 and multiply by 10.929

My proposition is that we should consider a different model for diabetes, one more clearly based around a molecular or pathophysiological taxonomy of diabetes; I call this the ‘palette’ model. The analogy here is with the painter who mixes a series of primary (and other) base colours to achieve an unlimited spectrum of hues and saturations (see Fig. 1 and Table 1).

In this model, the ‘base colours’ are the set of pathophysiological traits and processes (‘component pathways’) that contribute directly to the development of diabetes. It is simple to fashion a list of potential component pathways based on current knowledge; it would feature processes such as islet development, senescence and replacement (leading to variation in islet and beta cell number), islet function, islet autoimmunity, incretin activity, obesity, fat distribution and insulin resistance. This list is not meant to be exhaustive nor definitive; mapping the ontology and relationship of these processes is one of the challenges to be addressed.

For every one of these processes, each of us is positioned somewhere on a spectrum of phenotypic variation that feeds into our individual risk of type 2 diabetes. Much of the variation in each spectrum sits within what we would naturally consider the physiological range for that trait, in that measures in that range are perfectly compatible with entirely normal glucose tolerance. The position of an individual with respect to each process (by analogy, the depth of saturation for the particular hue that denotes that component) will be determined by the sum total of the relevant genetic variation that person has inherited and the personal history of the non-genetic exposures that influence that particular trait.

In turn, the overall position each of us occupies with respect to metabolic health and diabetes status represents, at any given point in time, a synthesis across all those individual traits. This is designated in Fig. 1 by a colour that reflects the mixture of the component-specific hues and saturations. If the integrated total of the trait values across multiple processes is sufficient to propel an individual to progress towards diabetes, the settings for many of these processes will be further adjusted by reactive responses to advancing hyperglycaemia (for example, further reductions in insulin secretion as a result of glucotoxicity).

## Why might the palette model be a more faithful way of mapping the pathogenesis of diabetes?

First, the palette model is clearly consistent with the current understanding of type 2 diabetes as a multisystem disease with evident abnormalities in the pancreas, fat, muscle, liver and, most likely, the brain.

Second, this approach is supported by the emerging genetic data. Amongst the >150 common variant signals for diabetes (all types) identified in genome-wide association studies (GWAS), there are examples of loci that influence each of the following processes: HLA loci for autoimmunity, *HNF1A* and *WFS1* for islet development, *CDKN2A* for islet senescence, *KLF14* and *PPARG* for adipogenesis, *FTO* for obesity, *KCNJ11* for islet function and so on [15, 16]. Carriers of risk alleles for each of these association signals are shifted a little rightwards in the respective trait spectrum, towards higher saturation (see Fig. 1). However, irrespective of the process directly involved, the consequence is only a subtle increase in diabetes risk. With better regulatory maps for key diabetes-relevant tissues, it is now possible to tie these genetic variants to tissue-specific effects on component pathways, not only on the basis of their impact on whole-body physiology [16], but also through their relationships with tissue-specific patterns of genome regulation and gene transcription [17]. For example, the type 2 diabetes GWAS-identified variant near *KLF14* is now known to exert its effects exclusively via the regulation of gene transcription in adipose tissue [18].

Third, this view of diabetes pathogenesis is consistent with the growing portfolio of available therapies. We have agents and interventions that can prevent or ameliorate diabetes through, for example, beneficial effects on islet function (e.g. sulfonylureas), obesity (weight loss), insulin resistance (e.g. exercise), fuel partitioning (e.g. thiazolidinediones) and microbiome content (metformin, possibly). Just as diabetes risk alleles influence metabolic phenotype through pushing individual positions on particular component spectra to the right, these interventions drive them to the left, towards a reduction in diabetes risk (see Fig. 1). The fact that most patients with diabetes respond to most treatments (admittedly to different degrees) irrespective of the specific processes through which they act fits the notion that diabetes represents the aggregate effect of multiple parallel contributions to disease. It should be noted, however, that type 1 diabetes is a special case in this regard: the absolute and permanent loss of insulin secretion clearly ‘breaks’ the system and limits the potential for beneficial interventions in other components to compensate and return the patient towards metabolic health.

As a framework for the taxonomy of diabetes, this model has greater compatibility with clinical observation and, in particular, the graded heterogeneity of the diabetic phenotype. It imagines individuals (both those with and without diabetes) positioned within a multidimensional space (reduced to two dimensions in Fig. 1, for illustrative purposes), the axes of which reflect the various processes that contribute to diabetes pathogenesis. For most people with diabetes (such as individual ‘c’ in Fig. 1), diabetes is not the consequence of marked failure in any one process; instead it results from the aggregate impact of several contributions to risk, any one of which may be entirely unremarkable in isolation. We could imagine that individual ‘c’ has a genetic risk profile and a history of life-course exposures that means they are overweight with a somewhat adverse distribution of excess fat. In conjunction with a modest reduction in insulin secretory reserve, reflecting a below-average complement of slightly underperforming beta cells, this configuration of metabolic characteristics suffices to drive that person towards diabetes. In the palette analogy, such an individual would end up a nondescript shade of taupe (between grey and brown), reflecting the concomitant contribution from multiple pathogenetic processes.

In contrast, there will be some individuals with diabetes for whom one of the component pathways has played a predominant role in the development of disease. Most obviously, this applies to those with monogenic disease (MODY, neonatal diabetes, lipodystrophies). But even in those families in which high-impact ‘causal’ alleles may be segregating, individual phenotypes will remain subject to the usual population variation in diabetes-related genetic risk and environmental exposures, introducing opportunities for phenotypic heterogeneity. Nevertheless, we would still expect such individuals, by virtue of their high-impact alleles, to cluster near the edges and corners of the multidimensional space. This would also be true for those with type 1 diabetes (especially early-onset type 1 diabetes), in whom there is a dominant contribution of islet autoimmunity. Other individuals with ‘asymmetric’ patterns of exposure across diabetes component pathways may also gravitate to the corners and edges of this space, such as those with extreme obesity perhaps, or those with ‘lean type 2 diabetes’ (in whom beta cell insufficiency is likely to be particularly influential). In the palette analogy, such individuals would be represented by a ‘purer’ hue (for example, individual ‘a’ in Fig. 1).

This model, which is based around a pathophysiological taxonomy, lends itself to the generation of a diagnostic classification system that, for the subset of individuals who map to the edges and corners, is superficially compatible with the pigeonhole model. At the same time however, through explicit recognition that for most people with diabetes multiple parallel processes (both primary and secondary) have contributed to disease development, the palette model makes it clear that aetiological complexity is not compatible with simplistic diagnostic categorisation.

A model based on a pathophysiological or molecular taxonomy, rather than rigid diagnostic classification, also has the advantage of more easily encompassing a dynamic, life-course view of disease evolution. Using this approach, one should be able to plot an individual’s path through multidimensional space as they transition from health to diabetes (or not). In early life, that individual’s location in multidimensional ‘metabolic’ space would be dominated by the impact of their genetic profile and early life environment on the function of the various diabetes component processes. As the individual matures and ages, diverse environmental exposures (related to employment, medication or other external circumstances) may increasingly leave their mark. If the net effect of these is to nudge the individual away from a position of compensated homeostasis, they will start to track along a path that leads towards others with diabetes, along the way adding reactive effects to the phenotypic mix that are secondary to disease progression (e.g. islet glucotoxicity) or interventions (e.g. addition of statins).

## Charting future progress

How does this model advance our progress towards personalised medicine? One take-home message is that we may be better off concentrating research efforts, at least initially, on those groups of individuals who lie in the edges and corners of metabolic space. Those individuals may more often represent ‘archetypes’: sets of individuals for whom their diabetes (or diabetes predisposition) is a more consistent, simple and homogeneous reflection of perturbations in a limited number of pathophysiological processes. They are likely to represent only a small minority of those with diabetes but access to ever larger biobanks (e.g. UK Biobank) and healthcare data (via electronic medical records) makes it possible to start to define such subgroups using combinations of clinical, anthropometric, biochemical, genetic and immunological measures. By necessity, initial efforts will focus on measures that are already available. However, a growing number of relevant variables are widely captured including BMI, waist:hip ratio, genetic risk profiles, islet antibody status, levels of C-peptide and C-reactive protein and measures of liver function [19, 20]. Recent attempts to extend our understanding of the pathogenesis of diabetes by embracing novel data types, such as those regarding gut microbiome content and complex health record data [21, 22], serve to illustrate both the opportunities and the challenges of these efforts as the range of data sources expands.

Mechanistic and translational studies that focus on the characterisation of archetypes are likely to be more tractable. For each of the component pathways, we should seek to deepen our understanding of the molecular and physiological machinery responsible for homeostatic control, and of the specific genetic and environmental factors that ‘push’ individuals towards diabetes. We should aim to identify biomarkers that serve as robust readouts for each of those processes. We already have some examples of these (e.g. islet antibodies, urinary C-peptide) but access to increasingly powerful ‘omic’ readouts (transcriptomics, proteomics, metabolomics) brings the promise of others [21]. We should aim to determine the extent to which the various pharmacological and behavioural interventions that are available influence diabetes progression and management in the different archetype groups. In doing so, we will determine the extent to which we can expect to optimise prevention and therapy on the basis of this improved diagnostic precision. Alternatively, we may find that many treatments work fairly well irrespective of individual pathology, since, to reverse the diabetic phenotype, it may be sufficient to shift enough of the contributing pathways in a beneficial direction.

Crucially, as our portfolio of process-specific biomarkers and/or interventions increases, we can begin to explore their joint performance in individuals with a more complex, ‘multiprocess’ disease. In other words, we can hope to move away from those in the edges and corners of multidimensional metabolic space and apply what we have learned to those individuals (likely the majority) who lie in the centre.

This vision of personalised medicine for diabetes envisages regular ‘pan-omic’ health checks, providing biomarker readouts that allow each of us to be positioned with respect to the range of diabetes component pathways and for our movements through multidimensional space to be tracked over time. Positions or trajectories that are known to presage future metabolic decompensation can be identified, and appropriate process-specific interventions instituted, guided by evidence that establishes the benefit of targeting reversal (or at least stabilisation) of the specific mechanisms driving disease progression in a given individual.

Only time (and much intensive research) will tell whether or not this vision can be realised. There is, of course, no guarantee that we will reach an easy resolution in terms of defining the specific component pathways that contribute to diabetes risk (or whether we will enter some recursive ‘Russian-doll’ world of ever more precise subdivision). Nor can we be sure that there will exist suitably sensitive and process-specific biomarkers, or targeted interventions for each of these. The routes by which individuals travel from health to disease are clearly varied and inherently complex; the hope is that a model that recognises and embraces this complexity will take us further than models that offer the illusion of diagnostic simplicity.

## Electronic supplementary material

Below is the link to the electronic supplementary material.

ESM 1 (PPTX 140 kb)

## Abbreviation

GWAS: Genome-wide association studies
