# Supplementary figures and images for: Painting a new picture of personalised medicine for diabetes
Source: Diabetologia. 2017 Feb 7;60(5):793–9. doi: 10.1007/s00125-017-4210-x (PMC6518376; doi:10.1007/s00125-017-4210-x)

## Slide 1
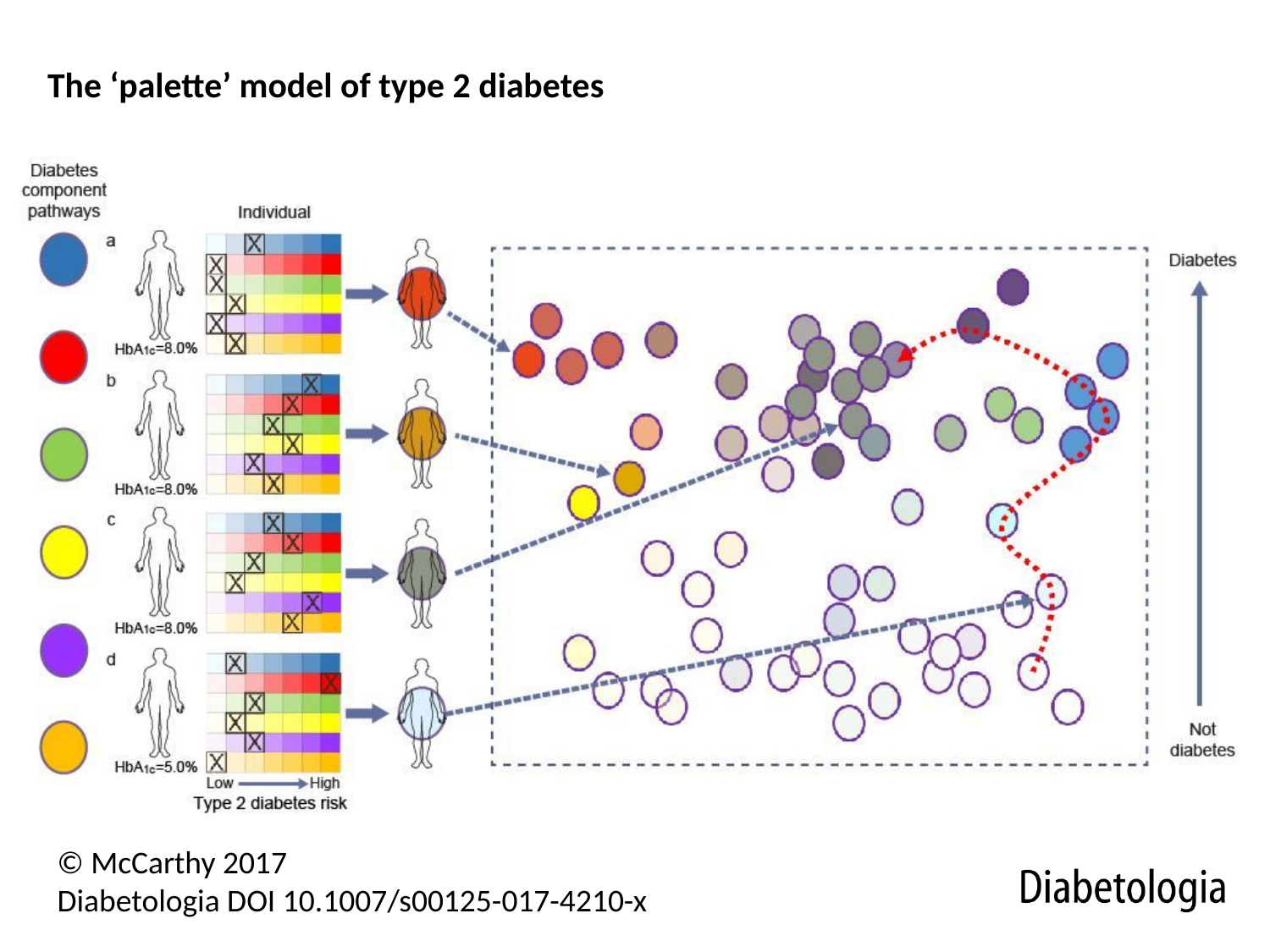

The ‘palette’ model of type 2 diabetes
© McCarthy 2017
Diabetologia DOI 10.1007/s00125-017-4210-x

Supplement: Supplementary file 1 — (PPTX 140 kb) [file 125_2017_4210_MOESM1_ESM.pptx]
